# Tracing Acetylene Dissolved in Transformer Oil by Tunable Diode Laser Absorption Spectrum

**DOI:** 10.1038/s41598-017-13823-0

**Published:** 2017-11-02

**Authors:** Guo-ming Ma, Shu-jing Zhao, Jun Jiang, Hong-tu Song, Cheng-rong Li, Ying-ting Luo, Hao Wu

**Affiliations:** 1 State Key Laboratory of Alternate Electrical Power System with Renewable Energy Sources, North China Electric Power University, Beijing, 102206 P. R. China; 2 College of Automation Engineering, Nanjing University of Aeronautics and Astronautics, Nanjing, 210016 China; 3 Electric Power Research Institute of Guangdong Power Grid Co., Ltd., Guangzhou, 510080 P. R. China

## Abstract

Dissolved gas analysis (DGA) is widely used in monitoring and diagnosing of power transformer, since the insulation material in the power transformer decomposes gases under abnormal operation condition. Among the gases, acetylene, as a symbol of low energy spark discharge and high energy electrical faults (arc discharge) of power transformer, is an important monitoring parameter. The current gas detection method used by the online DGA equipment suffers from problems such as cross sensitivity, electromagnetic compatibility and reliability. In this paper, an optical gas detection system based on TDLAS technology is proposed to detect acetylene dissolved in transformer oil. We selected a 1530.370 nm laser in the near infrared wavelength range to correspond to the absorption peak of acetylene, while using the wavelength modulation strategy and Herriott cell to improve the detection precision. Results show that the limit of detection reaches 0.49 ppm. The detection system responds quickly to changes of gas concentration and is easily to maintenance while has no electromagnetic interference, cross-sensitivity, or carrier gas. In addition, a complete detection process of the system takes only 8 minutes, implying a practical prospect of online monitoring technology.

## Introduction

Power transformers are important equipment in the power system, and the stable operation of the transformer is essential for power transmission. In order to prevent the transformer outage due to failure, it is important to monitoring the condition of the transformer^1,2^.

When specific defects such as overheating, partial discharge, or arc discharge occurs in the transformer, the insulation oil or paper decomposes and produces gases, and the gases are dissolved in insulating oil^3–6^. Then, the decomposition products in the oil reduce the insulation properties and change the interfacial tensor^3^. Thus, aging or defect information can be obtained by analyzing the volume, types, proportions, and rate of production of dissolved gases^7,8^.

The detection of dissolved gases in liquids has many important applications in different industries, such as water quality assessment (oxygen concentration in water), carbon dioxide concentration in carbonated beverages, and methane detection in crude oil. Besides that, dissolved gas analysis and detection in transformer oil is a useful method for evaluating the transformer condition^9–11^.

Dissolved gas analysis (DGA) is an oil-immersed transformer fault prediction and detection technique, based on the measurement of dissolved gases^12^. And there are some methods to detect fault gases, such as: gas chromatography (GC)^13^, thermal conductivity detector (TCD) and optical methods such as: spectral absorption method, photoacoustic spectroscopy^14,15^. However, these methods have following drawbacks:

1. GC is a widely used technique for quantitatively measuring fault gases concentration. Nevertheless, this analytical technique is not effective for on-line monitoring due to its large size, unstable in field and reliance on carrier gas and columns. After that, the chromatography analysis takes much time^16^. In addition, physical or chemical detectors are susceptible to cross-sensitivity, while electromagnetic interference and reliability also affect detection accuracy^17^.
2. The mechanical vibration and noise signals in the substation seriously affect the normal photoacoustic detection.
3. The main drawback of the direct absorption spectroscopy method is that it measures tiny signals at the top of the large background signal. Thus, any noise introduced by the light source or system reduce the detectability of the technology. Therefore, the sensitivity should be improved for field application.
4. Laser calorimetry spectroscopy (LCS), an in-liquid detection technique, will be a promising technology if it can achieve a lower detection limit, but at present, the minimum detection limit for acetylene in transformer oil is 10 ppm^18^ which does not meet the requirement of DGA.

The direct absorption spectroscopy can be improved in two aspects; to reduce the noise in the signal and to increase the absorption. And modulation techniques can reduce noise. Acetylene is produced in low energy spark discharge and high energy electrical faults (arc discharge). And acetylene detection is of great importance to monitor the incipient faults. Both Ding *et al*. and He *et al*. use tunable diode laser absorption spectroscopy (TDLAS) technology to detect acetylene gas, but their limit of detection do not meet the requirements for transformer condition monitoring^19,20^. Moreover, their experiments lack the discussion of the precision of the test. Thus, there is still a gap from the practical application. Deng *et al*. used Herriott cell to increase the optical path and detect acetylene gas, but these achievements are made in the gas conditions, unsuitable for transformer oil dissolved gas detection^21^.

There are some problems need to be solved if we want to use TDLAS technology and Herriott cell for gas detection in transformer oil. For example, if the output laser is directly irradiated in the transformer oil, most of the light intensity will be absorbed by the oil, so the gas cannot be detected. If we increase the optical path to improve the absorption of gas, according to our test results, when the oil sample thickness of 4 cm, only 52.3% of the light intensity through the oil sample, for wavelength of 1557 nm. To solve the problems, a new method is proposed in the paper.

Herriott cell cannot be directly used in oil immersion conditions, since the insulating oil pollutes the mirrors in Herriott cell. Thus, we designed a separation device, using the vacuum degassing method to determine the degassing efficiency, and carried out the calibration test. In the manuscript, the results of the calibration test show that TDLAS technology and Herriott cell can be used to detect dissolved gases in the oil using our designed equipment.

In our case, we modulate the output wavelength of the laser. Near infrared DFB lasers are used in manuscript, for it has an economical advantage over mid-infrared lasers. And the detection sensitivity of the proposed measuring system is also satisfied. The experimental device designed in the manuscript uses Herriott cell and wavelength modulation to improve the monitoring sensitivity, and to meet the requirements of online testing.

## Theory of TDLAS technology

When a beam of light passes through an absorption cell with a certain concentration of gas, the light intensity will be attenuated to some extent. This phenomenon reflects the change in gas concentration, and the gas concentration can be determined by measuring the intensity attenuation, which is called Beer-Lambert’s law.

Beer-Lambert Law can be expressed as:

1 $$I({v})={{\rm{I}}}_{0}\times \exp [-\alpha (v)\times {\rm{C}}\times {\rm{L}}]$$

where I (v) and I0 (v) are intensities of incident and emitted laser respectively, V. C is the concentration of gas to be detected, ppm. L is the path length of the beam of light through the material sample, m. α(*v*) is the absorption coefficient per unit distance and per unit concentration of the gas, cm/mol. A is absorbance.

2 $${\rm{A}}=-\mathrm{ln}(I({v})/{{\rm{I}}}_{0}(v))=\alpha (v)\times {\rm{C}}\times {\rm{L}}$$

3 $$\frac{{d}^{n}{\rm{A}}}{d{v}^{n}}=\frac{{d}^{n}\,\alpha (v)}{d{v}^{n}}\times {\rm{C}}\times {\rm{L}}$$

The derivative of A shows a linear relationship between the amplitude and the concentration C as other conditions do not change. When n = 2, the 2 f signal is

$$\frac{{d}^{2}{\rm{A}}}{d{v}^{2}}=\frac{{d}^{2}\,\alpha (v)}{d{v}^{2}}\times {\rm{C}}\times {\rm{L}}$$

**Figure 1:**
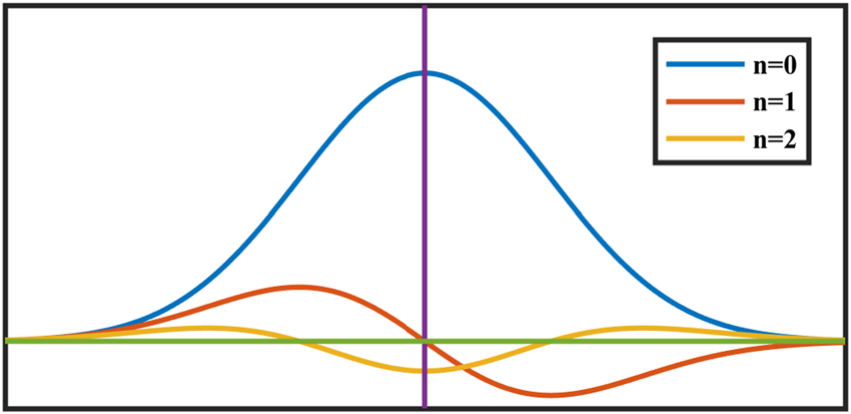
The different order derivatives of absorbance line shape. The 2f signal shape is drawn as a yellow line.

The different order derivatives of A are shown in Fig. 1.

**Figure 2:**
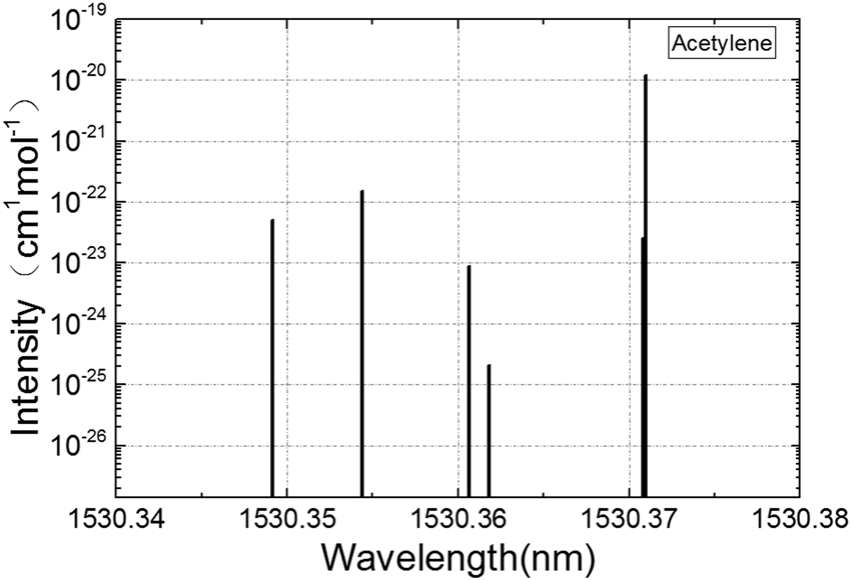
Acetylene gas molecule absorption intensity distribution in the range of 1530.34 ~ 1530.38 nm (from HITRAN2012 database). Absorption intensity is also referred as line strength, and the unit is cm^–1^/(molecule/cm^2^) in HITRAN database.

According to HITRAN database, acetylene has 5560 absorption lines in the near infrared band (780 nm ~ 2526 nm. When it comes to absorption lines, the high absorption intensity and the avoidance of interference are the main concerns. For these considerations, we chose the “P9 peak” of acetylene (1530.370 nm) as the central wavelength of the DFB laser. The molecule absorption intensity distribution of acetylene is shown in Fig. 2, available from website http://hitran.org/.

TDLAS uses a laser with a line width much smaller than the gas absorption line width, and a single absorption line of the gas is scanned by wavelength modulation^22,23^. Then, we can obtain the harmonic component of the absorption signal through the lock-in amplifier, and the concentration of the test gas is proportional to the amplitude of the harmonic signal.

The coefficient of the odd harmonic component is zero at the center wavelength and the even harmonic is the maximum. Since the amplitude of the harmonic signal decreases with increasing order, we choose the second harmonic as the actual detection signal. As a calibration experiment is carried out to determine the relationship between the gas concentration and the second harmonic signal amplitude, we can measure the harmonic signal to obtain gas concentration in field application^24^.

## Experimental setup

The test platform is mainly composed of oil chamber, gas detection system and vacuum device. Among them, the oil chamber is designed to complete the oil and gas separation. The gas to be measured then passed into the air chamber and the acetylene concentration information is detected by the TDLAS controller and the acquisition system. The vacuum pump is used to make the vacuum of the gas detection system and to remove the impurity gas (including the last test residual gas).

Gas preparation method is as follows: Use a syringe to take 40 mL of oil, inject 5 mL of acetylene gas and seal it. Place the syringes on a vibrating and heating platform for oil-gas mixing. The heating temperature is set to be 50 degrees Celsius while the vibration lasts half hour. Take a few drops from the syringes to the beaker, then add different volume pure transformer oil to obtain the different concentrations test oil samples.

**Figure 3:**
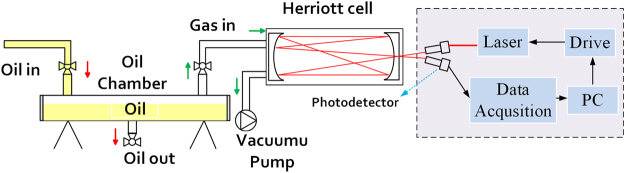
Experimental setup of TDLAS measurement. The oil chamber in the experimental setup is used for oil-gas separation. Herriott cell is used to increase the optical path. Control and measurement system is used to wavelength modulation and data measurement.

Seven groups of different concentrations of oil were prepared in the test, about 500 mL in each group, of which 300 mL was used for oil-gas separation and TDLAS testing, and 40 mL was used for gas chromatography. The gas detection system uses a multi-pass Herriott cell, which has the advantages of small size (34 cm), long optical path length (10.1 m), strong absorbance and high sensitivity. The experimental setup is shown in Fig. 3. The photodetector used by measuring system was encapsulated in metal shield, which has good electromagnetic compatibility.

The test process involves two types of oil samples: pure oil and dissolved acetylene oil. The test should be in accordance with the following steps:

- Test sequence: pure oil → dissolved acetylene oil (from low to high concentration).
- Ensure the oil chamber and air chamber air tightness.
- When the transformer oil enters and avoid polluting the air chamber, slowly open the exhaust valve within 1 minute.
- Before vacuum extraction, oil sample is prepared for offline DGA test in the lab for 3 times and the average value is recorded.

**Figure 4:**
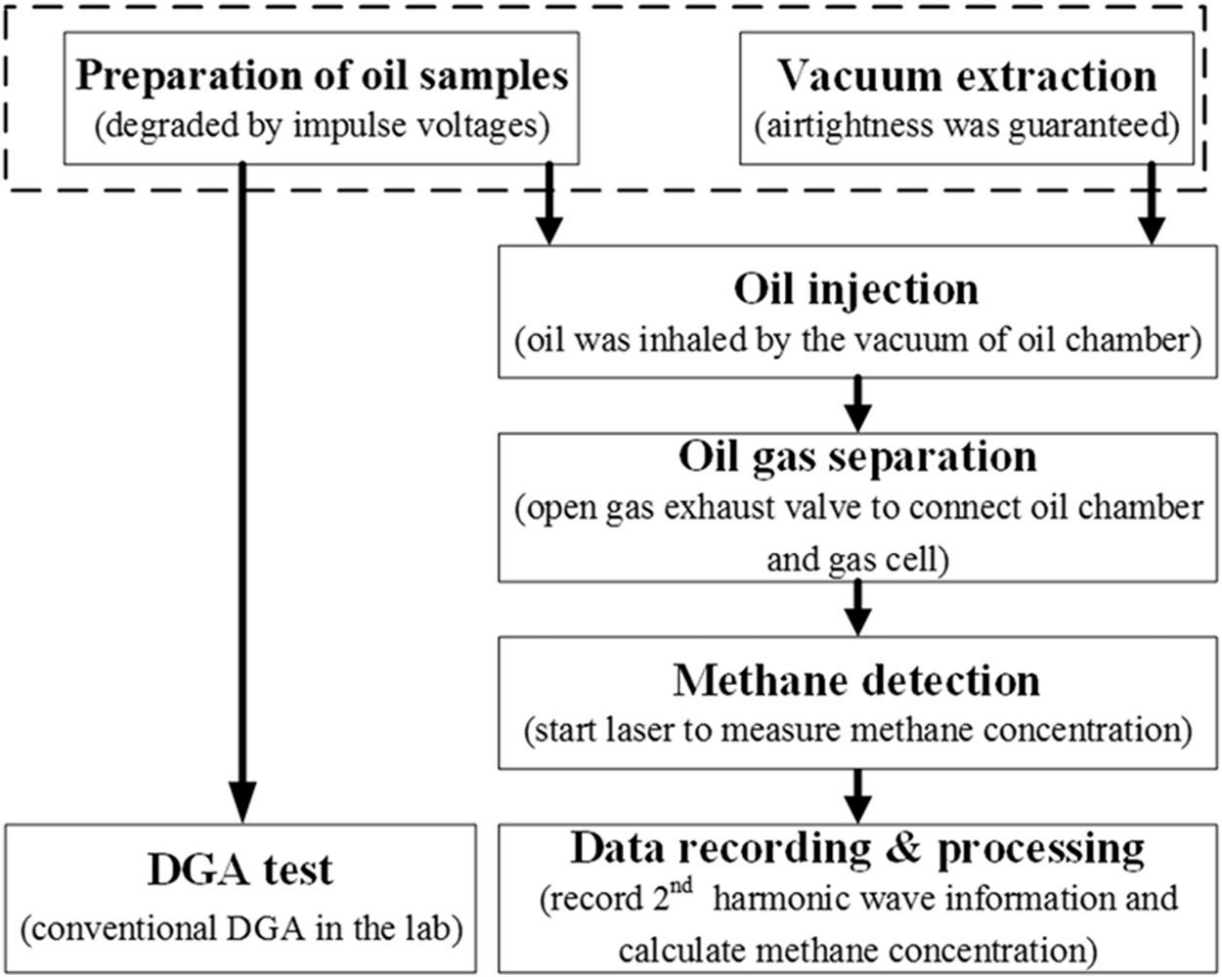
Procedures of measurement.

The main procedure of TDLAS detection includes oil sample preparation, vacuum extraction, oil injection, oil and gas separation, acetylene detection and data recording and processing, as shown in Fig. 4. Detection process does not require carrier gas. After oil and gas separation, the transformer oil by degassing, filtration can be injected back to the transformer.

It should be noted that the operating time of the TDLAS gas detection system includes the following steps: vacuum extraction, 2 minute; oil injection, 1 minute; oil and gas separation, 2 min; gas detection and data processing, 1 minute; emptying the oil chamber, 2 min. Therefore, the entire detection process is less than 8 minutes which can meet the needs of on-line monitoring and rapid measurement.

The detection system can respond quickly to changes in gas concentration. When the oil and gas separation is complete, the detection system can get the gas signal within 5 seconds.

## Results

### Cross-sensitivity

Typically, there are 7 fault gases produced by oxidation, insulation decomposition, and oil breakdown: H_2_, CH_4_, C_2_H_2_, C_2_H_4_, C_2_H_6_, CO, CO_2_. H_2_O is also included in the oil.

**Figure 5:**
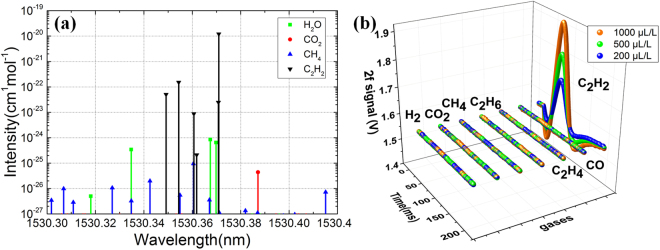
(**a**) The absorption line of the gases and moisture that may dissolve in the transformer oil (**b**) 2 f signal of seven gases (C_2_H_2_, H_2_, CO_2_, CH_4_, C_2_H_6_, C_2_H_4_ and CO) in gas phase, the carrier gas is nitrogen.

In order to determine whether there is a multi-gas cross-sensitive problem in the selected wavelength range, we query the HITRAN database and perform cross-sensitivity testing. The absence of absorption lines of CH_4_, C_2_H_2_, H_2_O, and CO_2_ from HITRAN in the vicinity of 1530.370 nm are shown in Fig. 5(a). In the region, water (other impurities absorption intensity is weak) has absorption line, but the absorption intensity of water is four orders of magnitude lower than that of acetylene. Besides that, the water content in the oil is strictly controlled to very small for insulation strength. The impact of these impurities on the detection of acetylene is minimal. The gas listed in Fig. 5(b) is the fault gas that characterizes the transformer failure. It can be seen that these impurities do not affect acetylene detection.

Therefore, the selection of wavelengths of 1530.370 nm absorption lines can avoid H_2_, CH_4_, C_2_H_4_, C_2_H_6_, CO, CO_2_ gas cross-interference.

In the same environment, different concentrations of C_2_H_2_, H_2_, CO_2_, CH_4_, C_2_H_6_, C_2_H_4_ and CO, ranging from 200 to 1000 ppm were mixed and measured, and 2 f signals were shown in Fig. 5(b), where only acetylene gas has a significant absorption signal, the absorption of other gases does not exceed 2% of acetylene.

The results of cross sensitivity test showed that H_2_, CH_4_, C_2_H_4_, C_2_H_6_, CO, and CO_2_ do not change with the increase of concentration in the selected wavelength range. Only the acetylene gas concentration and the second harmonic signal showed a significant positive correlation. Concluded from the absorption intensity in the HITRAN database and the result of multi gases interference tests, the detection of acetylene in this wavelength range is not affected by the concentration of other gases.

### Result of calibration

According to the procedure mentioned in Fig. 4, the prepared oil samples were injected into the oil chamber. The detection performance indexes of the TDLAS system are obtained with experiment, including goodness of fit, repeatability, limit of detection.

**Figure 6:**
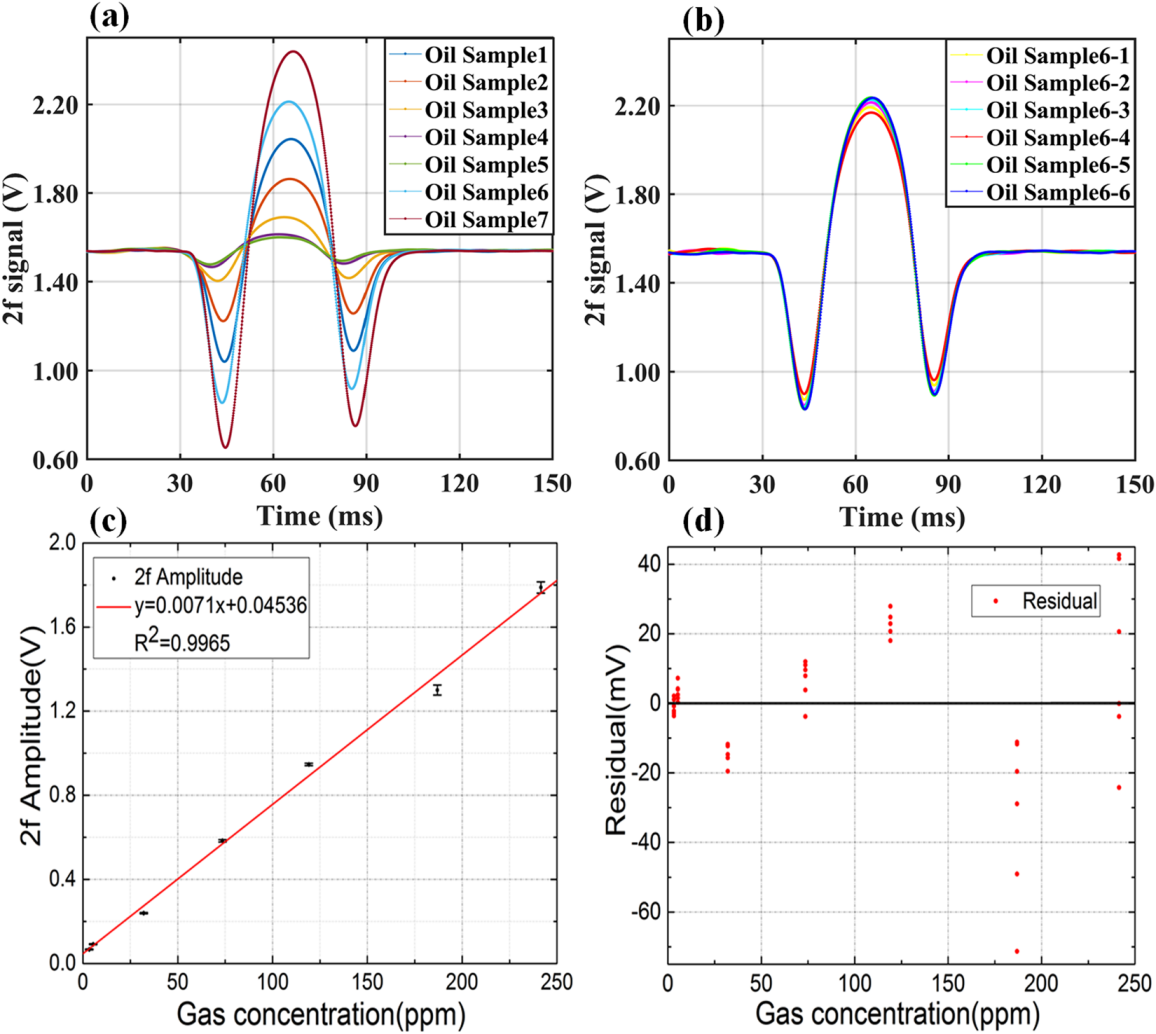
(**a**) 2f signals of seven different concentrations acetylene of oil in the calibration test. (**b**) 2 f signals of acetylene dissolved in similar concentration oil samples. (**c**) Fitting curve of 2 f signal and dissolved acetylene concentration in transformer oil. (**d**) Residual error of linear fitting.

There were seven different concentrations acetylene of oil in the test. Six oil samples were prepared for each concentration. We tested a total of 42 oil samples and shown the test result at Fig. 6. Equation (4) is calculated based on the actual measurement of all 42 oil samples.

#### Goodness of fit

The test results show that 2 f signal is proportional to the concentration of acetylene gas dissolved in the oil as shown in Fig. 6(c). According to 2 f signal and gas concentration to fit, the expression is:

4 $$y=0.0071\times x+0.0454$$

*x* is the concentration of gas to be detected, ppm. *y* is the 2 f signal amplitude, V.

The coefficient of determination R^2^ is 0.9965. A linear relationship between peak-peak value of 2 f signal and acetylene concentration can be obtained. And the sensitivity can be calculated as 7.1 mV/ppm.

To investigate the goodness of fitting, the amplitude of 2 f signal was substituted into fitting equation, and residual errors were obtained by subtracting calculated value from conventional DGA results, as shown in Fig. 6(d).

#### Repeatability

In the test, all seven different concentrations oil samples were tested for six times.

No. 6 concentration oil samples are the most dispersed. No.6 oil samples concentrations is 186.9 ppm, measured by GC (gas chromatography). The average concentration of the six tests is 176.6 ppm. The deviation is 5.6% of the value measured by GC.

The system has better performance in lower concentration range (below 50 ppm).The maximum concentration deviation is 1.4 ppm.

The results show that the trace gas measurement system has good repeatability.

#### Limit of detection

**Figure 7:**
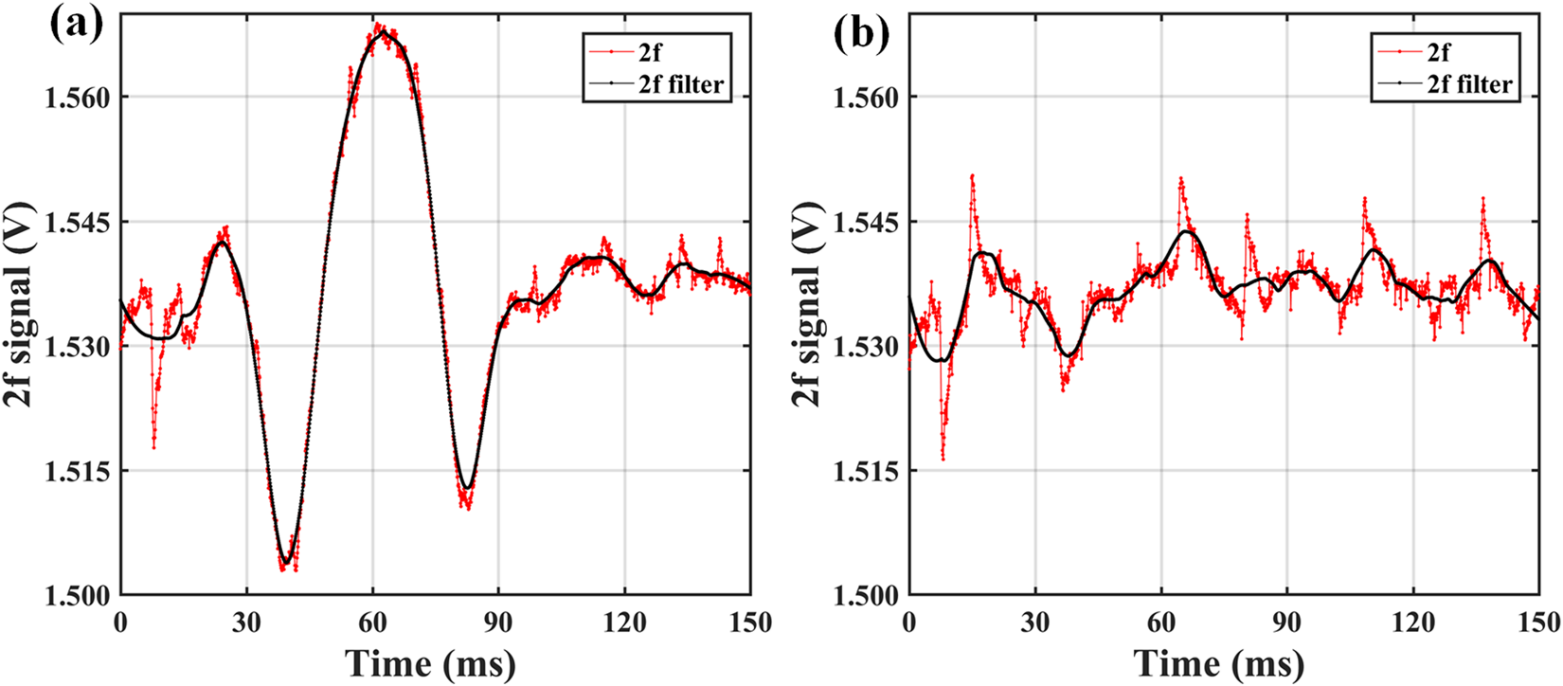
Initial and pre-processed 2 f signal of oil sample. (**a**) A signal with acetylene concentration of 3.25 ppm (**b**) Noise signal.

In order to obtain limit of detection, a signal-noise ratio (SNR) method is commonly used. It is generally considered that the signal-to-noise ratio at 3: 1 is acceptable for the limit of detection. The initial noise data that not been pre-processed has a large effect on the detection limit, so we use Savitzky–Golay filter^25,26^ to reduce the effect of high frequency noise on the calculation results. The results of the filtering are shown in Fig. 7.

Then the blank noise is 0.0163 V, and the limit of detection is:

5 $$(3\times 0.0163-0.0454)/0.0071=0.49\,\mathrm{ppm}$$

### Test results of operational transformer

We obtained four oil samples from two operational transformers in the 220 kV substation. The test results are shown in Table 1. Oil samples were taken from Gukeng substation 220 kV transformer, Dongguan City, Guangdong Province, China. For the transformer, high concentration of acetylene indicates that the transformer has some defects that may induce transformer failure, and low concentration we measured indicates that the transformer is operating in good condition.

**Table 1:** Test results of operational transformer.

Acetylene	Test results by proposed system (ppm)	Test results by GC (ppm)	Deviation (ppm)
Oil Sample 1	1.17	0.62	0.55
Oil Sample 2	1.42	0.81	0.61
Oil Sample 3	1.25	0.66	0.59
Oil Sample 4	0.95	1.08	−0.13

The maximum measurement deviation of the test results is 0.61 ppm. These results indicate that the detection system designed in this manuscript can be used for substation monitoring.

According to standard IEC 60599 and IEEE Guide for the Interpretation of Gases Generated in Oil-Immersed Transformers, acetylene ranges of 90% typical gas concentration values observed in power transformers, from about 25 electrical networks worldwide and including more than 20000 transformers, is 2–20ppm. And a four-level criterion has been developed to classify risks to transformers. For condition 4, when acetylene > 35ppm, continued operation could result in failure of the transformer. Thus, the detection range of the system in the experiment, 0–250 ppm, is enough to evaluate the system performance.

## Conclusion

In this paper, the following conclusions have been drawn

I. A new optical method based on the tunable diode laser absorption spectroscopy (TDLAS) technique is proposed to detect the dissolved acetylene gas in transformer oil. The wavelength modulation strategy with center wavelength of 1530.370 nm and Herriott cell were selected to ensure high sensitivity detection. The TDLAS acetylene detection system developed in this manuscript has excellent performance: sensitivity of 7.1 mV /ppm; limit of detection is 0.49 ppm, lower than the online monitoring requirement.
II. The proposed detection system has tremendous advantages, such as: immediate response to concentration changes; eliminating carrier gas; no cross interference from background gases; wide range of measurable gases. And the system can save the time of detection, with the detection process less than eight minutes, proved to be a promising technique instead of conventional DGA equipment.

### Data availability statement

All data generated or analyzed during this study are included in this published article.
